# Investigating Students’ Answering Behaviors in a Computer-Based Mathematics Algebra Test: A Cognitive-Load Perspective

**DOI:** 10.3390/bs12080293

**Published:** 2022-08-18

**Authors:** Jing-Fong Wang, Tzu-Hua Wang, Chao-Hsien Huang

**Affiliations:** 1 Department of Education and Learning Technology, National Tsing Hua University, Hsinchu 300193, Taiwan; 2 Research Center for Education and Mind Sciences, National Tsing Hua University, Hsinchu 300193, Taiwan

**Keywords:** computer-based testing, cognitive load, mathematics algebra questions, element interactivity, practice effect, individual differences

## Abstract

Computer-based testing is an emerging method to evaluate students’ mathematics learning outcomes. However, algebra problems impose a high cognitive load due to requiring multiple calculation steps, which might reduce students’ performance in computer-based testing. In order to understand students’ cognitive load when answering algebra questions in a computer-based testing environment, three perspectives, element interactivity, practice effect, and individual differences, were investigated in this study. Seven levels of algebra exam questions were created using unary and simultaneous linear equations, and the inverse efficiency scores were employed as a measure of cognitive load in the study. Forty undergraduate and graduate students were tested. There were four findings: (1) As the element interactivity of test materials increased, the cognitive load increased rapidly. (2) The high-efficiency group had a lower cognitive load than the low-efficiency group, suggesting that the high-efficiency group had an advantage in a computer-based testing environment. (3) “Practice” has a considerable effect on reducing cognitive load, particularly in level 6 and 7 test items. (4) The low-efficiency group can reduce but not eliminate the gap with the high-efficiency group; they may require additional experience in a computer-based testing environment in order to improve reducing their cognitive load.

## 1. Introduction

Computer-based testing is an emerging method adopted to evaluate student learning outcomes. Advances in technology have rendered computer-based testing more popular. Key drivers of this trend include the ability to provide various types of test items, automatic scoring, and a real-phase error reduction in computer-based testing, which enable immediate analysis and the provision of the required information as feedback for easy archiving and inquiry. Test items and a shared item database can also be easily generated [1,2]. Ideally, students’ computer- and paper-based testing performance should be the same [3,4]. However, computer-based testing differs from paper-based testing in that student learning outcomes may be underestimated in the former test type due to technical considerations [5,6]. Consequently, the reliability and validity of computer-based testing have been questioned by some researchers [7,8]. For example, in their review article, Wang, Jiao, Young, Brooks, and Olson (2007) conducted a meta-analysis of computer- and paper-based mathematics tests targeting K-12 students in the past two decades and observed inconsistency between the performance of the two test types [7]. Logan (2015) observed no significant differences in examinees’ performance between paper- and computer-based testing on mathematics [9], whereas Hensley (2015) reported significant differences [10]. Such inconsistency in performance may be ascribed to how computer-based testing changes the way examinees respond to test items [7]. That is, problems exist concerning differences in the item response process between paper- and computer-based testing [11]. In addition to the aforementioned technical issues, cognitive load is another factor that reduces students’ responses to test items, as proposed by Sweller et al. [12,13]. Cognitive load is divided into three types: intrinsic cognitive load, extraneous cognitive load, and germane cognitive load. The intrinsic cognitive load is related to the information and complexity of the learning materials; the extraneous cognitive load is related to the teaching method and is unrelated to learning material difficulty, and the germane cognitive load is related to how knowledge is acquired [14]. According to cognitive-load theory, the same test, when administered as a computer-based test, can affect the extraneous cognitive load and germane cognitive load in examinees, both of which affect test performance.

Chen, Woolcott, and Sweller (2017) mentioned that teachers must pay attention to cognitive load in computer-based learning to devise appropriate instructional designs [15]. When teaching mathematics to impart the logical thinking required for mathematical problem solving, teachers should consider the cognitive load of the learning material imposed on students because the cognitive load affects their learning outcome. Easy learning materials require fewer cognitive resources and lower the cognitive load imposed on learners. By contrast, difficult learning materials require more cognitive resources and impose a higher cognitive load on learners. Therefore, understanding the cognitive-load status of learners is necessary. The relationship between learning materials and cognitive resources can be examined from the perspective of element interactivity in cognitive-load theory, which advocates that the difficulty of learning materials is determined by the interactivity between elements [12,16,17,18]. For example, the equation “5 + 2 = 7” contains several elements: numbers (i.e., 5, 2, and 7) and symbols (i.e., + and =). Learners must consider more than one element (both numbers and symbols in the aforementioned example) while processing and integrating the information conveyed by the elements. For learning materials, high element interactivity between elements imposes a high intrinsic cognitive load, whereas low element interactivity imposes a low cognitive load. Overall, interactivity between the elements of the learning material increases with its difficulty, which imposes a high cognitive load on learners.

In mathematics education, algebra learning materials involve high interactivity of elements [19] because a variety of complex elements needs to be considered. This explains why algebra is considered a difficult subject in terms of evaluation through computer-based testing; in addition to the difficulty of test items, the problem of the split effect must be considered. In Mayer’s opinion, the digital media environment should avoid overloading the working memory capacity of visual and language channels [20,21]. In computer-based testing for mathematics, a common method is that students receive mathematics problems presented from a screen and calculate with a pen and paper; however, this process generates the split effect [22,23], which is considered an extraneous cognitive load. Thus, the split effect must be controlled and avoided to ensure the authenticity of scores of computer-based testing. The implementation of a rigorous test-taking mechanism (e.g., students may not use a pen and paper for calculation) can more accurately measure examinees’ cognitive ability and enable students to use visual channels for information processing, thereby reducing the interference of the split effect.

Based on the aforementioned discussions, two points need to be considered when implementing a computer-based mathematics algebra test: first, the split effect induced by computer-based testing administered to evaluate students’ mathematics knowledge may impose a cognitive load [24], which is called the extraneous cognitive load (ECL); second, the difficulty of algebra equations may also impose an intrinsic high cognitive load (ICL) on examinees in computer-based testing, which, in turn, affects examinees’ performance. The digital environment and algebra equations require students to apply certain amounts of cognitive resources, which means that working memory capacity may affect students’ mathematics performance [25].

On how to reduce the cognitive load of learners in a digital environment, Mayer and Moreno (2003) propose nine ways to solve the problem [26]. However, most of the above ways belong to the improvement in the presentation of materials and additional auxiliary methods provided by teachers, and the method that is driven by the learner’s own internal learning mechanism is less discussed—namely, the effect of practice. According to a research report, a practice not only enables the improvement in the efficiency of performing tasks but also reduces cognitive load effectively [27]. Individual differences are also an essential issue to consider when it comes to cognitive load. According to research on EFL students, individuals with low visual learning ability performed worse and had a larger cognitive load than those with high visual learning ability [28]. To summarize, individual differences and the relationships between other factors, such as the relationship between individual differences in cognitive load and the practice effect, should be considered.

### Aims of This Study

Regarding the differences in the response process of paper-based testing and computer-based testing, Lemmo (2021) contends that, even though students’ performance on paper-based testing and computer-based testing are consistent, they are not equivalent in the processing [29]. Also, Wang et al. (2021) explored students’ performance on computer-based testing and paper-based testing, and factors such as question difficulty and presentation order, which are related to the intrinsic and extraneous cognitive loads, respectively [30]. Additionally, computer-based testing has garnered a lot of attention during the COVID-19 pandemic, when in-person evaluations are not available [30]. Given the aforementioned, computer-based testing is the primary focus of this study.

Based on Sweller’s cognitive-load theory [13,14,16] and Mayer’s insights into learning in a computer-based testing environment [22,25], we conclude that the administration of digital examinations on algebra entails two major cognitive loads: (1) an extraneous cognitive load induced by the split effect of the test format—namely, examinees need to face the display of test items on the screen and the use of a pen and paper for calculation at the same phase—and (2) the difficulty of the material itself, which is considered an intrinsic cognitive load. In this study, participants took the tests without a pen and paper to control the split effect, and the intrinsic cognitive load derived from the difficulty of mathematics materials was used as the basis of the experimental design. In addition to investigating the cognitive load caused by the difficulty of learning materials in a computer-based testing environment, individual differences and the practice effect were also investigated in this study. The following are the four major questions:

1. Would the cognitive load increase rapidly or slowly as the material’s element interactivity increases?
2. Would the high-efficiency group have the advantage of resisting the impact in the computer-based testing environment (e.g., without the aid of paper and pen)?
3. Would “practice” have an influence on the cognitive load of individuals?
4. Considering individual differences in “efficiency”, would the cognitive load disparity between the high- and low-efficiency groups be reduced or equalized through practice?

## 2. Methodology

### 2.1. Participants

This study recruited 40 college and graduate students, half of whom were men, and the mean age of the participants was 24 years. All students had learned unary and simultaneous linear equations. All the participants were healthy adults (without cognitive impairment), and, at the time of recruitment, it was stated that the mathematics item measured in this study was in the range of grades 5–7. Ultimately, half of the students came from the faculty of arts or education, and the other half from the faculty of science or engineering. 

### 2.2. Materials

In this study, the number of calculation steps served as the standard for setting the difficulty of test items. Test items consisting of unary and simultaneous linear equations were divided into seven levels of difficulty. Items of each level corresponded to a certain number of calculation steps; the higher the level of test items, the more the calculation steps required, and the higher the difficulty of test items. 

Examples of calculation steps for solving unary and simultaneous linear equations are as follows:

(1) The example of a unary linear equation (one calculation step):
  X + 5 = 9,
  The calculation procedure is:

Step 1: transposition: X = 9 − 5

(2) The example of simultaneous linear equations (five calculation steps):
  $$\{\begin{array}{c} x+3y=34\;\text{-}①\\ 5x+3y=62\;\text{-}② \end{array}$$
  The calculation procedure is:

Step 1: subtraction ② − ①: 4x = 28

Step 2: divide both sides by 4: x = 7

Step 3: substitute x = 7 into ①: 7 + 3y = 34

Step 4: transposition: 3y = 34 − 7 = 27

Step 5: divide both sides by 3: y = 9

Table 1 details the information on seven levels of difficulty designed in this study.

### 2.3. Data Collection 

The computer-based test, a common behavior measurement approach, was used in this study not only to record the accuracy and response time of students but also to set a precise time limit for test-takers to answer each test item; thus, learners were required to complete the test under a time constraint. This study used inverse efficiency scores (IES) for data analysis, which are calculated as follows:

IES = RT/PC,(1)

where RT denotes the mean response time, and PC is the percentage of correct answers.

**Figure 1:**
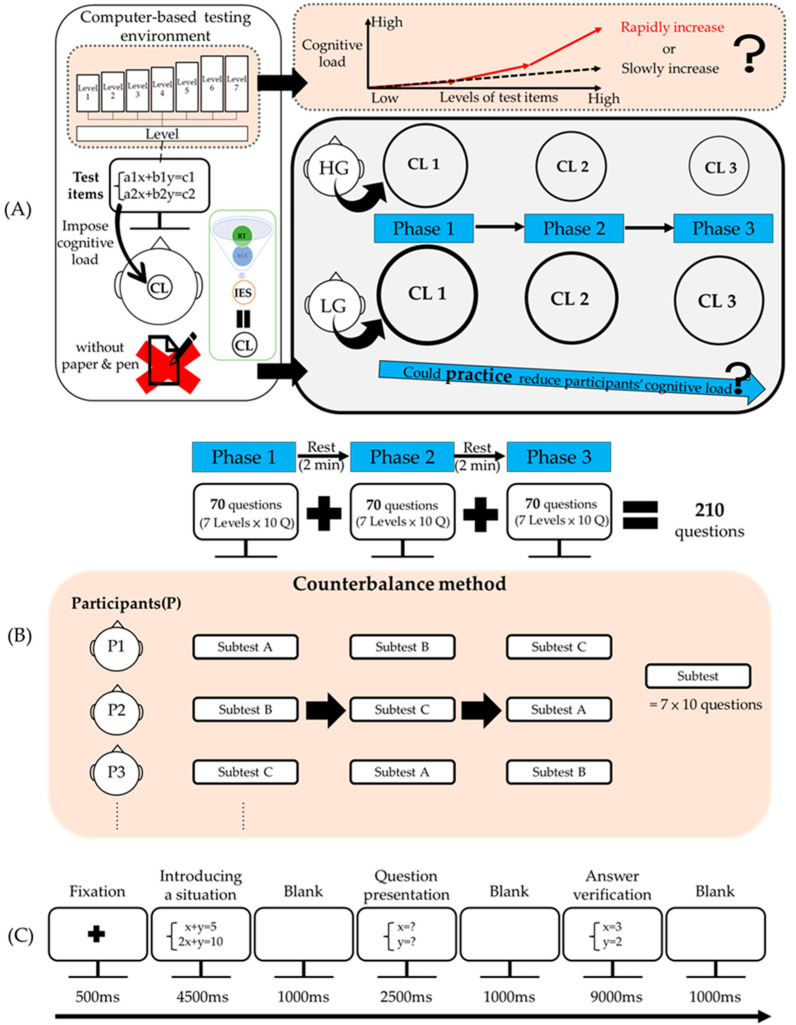
Research process. (**A**) Research structure. HG/LG refers to high- and low-efficiency. groups. CL refers to cognitive load. (**B**) The test administration procedure was divided into three phases. A counterbalance method was adopted to determine the order in which subtests should be administered. (**C**) The procedure for each test item.

Accuracy and response time are two indicators often used to measure cognitive ability in behavioral research, and each indicator has its connotation. Accuracy (the percentage of correct answers) indicates whether a test item is easy to answer, and the response time (how fast/slow a test-taker submits the answer) reflects the difficulty of a test item. Occasionally, participants respond rapidly at the expense of reduced accuracy or slowly to achieve higher accuracy. The aforementioned two situations cannot be avoided if either of the two indicators is used. Therefore, an indicator combining accuracy and response time was developed and termed IES, which is commonly applied to maintain a balance between accuracy and response time [31,32]; this indicator can more accurately reflect cognitive ability. Because the cognitive load affects cognitive ability, the cognitive process of students exposed to a high cognitive load in the learning process (i.e., high interactivity between elements of the learning material) is negatively influenced, resulting in an increased burden on working memory [14]. Working memory usually correlates with higher-order cognitive abilities in healthy adults [33]. This study used IES as the indicator of cognitive load to evaluate students’ test performance. Furthermore, we averaged the inverse efficiency scores (IES) of 7 different levels (Figure 1A) to determine the high- and low-efficiency groups, with the top 50% of the average IES indicating high efficiency and the bottom 50% indicating low efficiency. It is important to note that IES is inversely proportional to efficiency: the better the efficiency, the lower the IES.

### 2.4. Research Process and Data Analysis

Information on this study was disseminated online to recruit volunteers. After the informed consent of the participants was obtained, they took a 1.5-hour test, after which they were rewarded TWD 200. This study was reviewed and approved by a research ethics committee.

In this study, a mixed three-factor experimental design was adopted. Figure 1A depicts the research framework for the current investigation. Students in a rigorous computer-based testing environment (without paper and pen) were shown in the left box, while the upper right box (orange) inquired if their cognitive load increased in a rapid or slow pattern as the difficulty of test items increased. We assume that when participants are faced with simple problems, the cognitive load of those who are in a state of low cognitive load should increase slowly; nevertheless, when they were faced with difficult problems and had insufficient cognitive capacity to deal with them, there will be a rapid sharp increase, and the increasing rate will be much larger than the previous increasing trend—this is defined as a rapidly increasing pattern in this study. In other words, when facing a series of questions, by observing the increasing pattern of cognitive load, we can obtain a rough idea of the possible critical point of cognitive load. The right box showed that different efficiency (high- and low-efficiency) groups would have varying cognitive loads (notice the circle), and inquired if their cognitive load decreased as their practice increased.

Figure 1B presents the test administration procedure of the current study. Test items were administered in three phases and in sequential order. In each phase, one of the three subtests was administered, and a counterbalance method was adopted to determine the order in which the subtests should be administered. A 2-minute interval was allowed between phases. All participants completed the three subtests, and 70 test items—10 for each level of difficulty—were randomly displayed in each subtest. 

Figure 1C exemplifies the procedure for each test item. The procedure entails the following steps: 0.5 s of fixation, 4.5 s of introducing a situation, 1 s of blank, 2.5 s of question presentation, 1 s of blank, and, lastly, 9 s of “yes or no” responses when a solution appears (answer verification).

To conduct the analysis, we used a mixed three-way ANOVA to respond to the first three main questions and a mixed two-way ANOVA to respond to the fourth major question. In mixed three-way ANOVA, the Level factor was built up to comprehend the pattern of rising cognitive load, and the Phase factor with three test time points was set up to study the practice effect; finally, the Group factor with high- and low-efficiency groups was established by using the inverse efficiency score (IES). In addition, in the mixed two-way ANOVA (Level and Phase), the first phase of the high-efficiency group and the third phase of the low-efficiency group were used as the Group factor to analyze.

## 3. Results

Section 3.1 provides a mixed three-way ANOVA to respond to the major questions 1–3, and Section 3.2 provides a mixed two-way ANOVA to respond to major question 4.

### 3.1. A Mixed Three-Way ANOVA (G × L × P)

**Figure 2:**
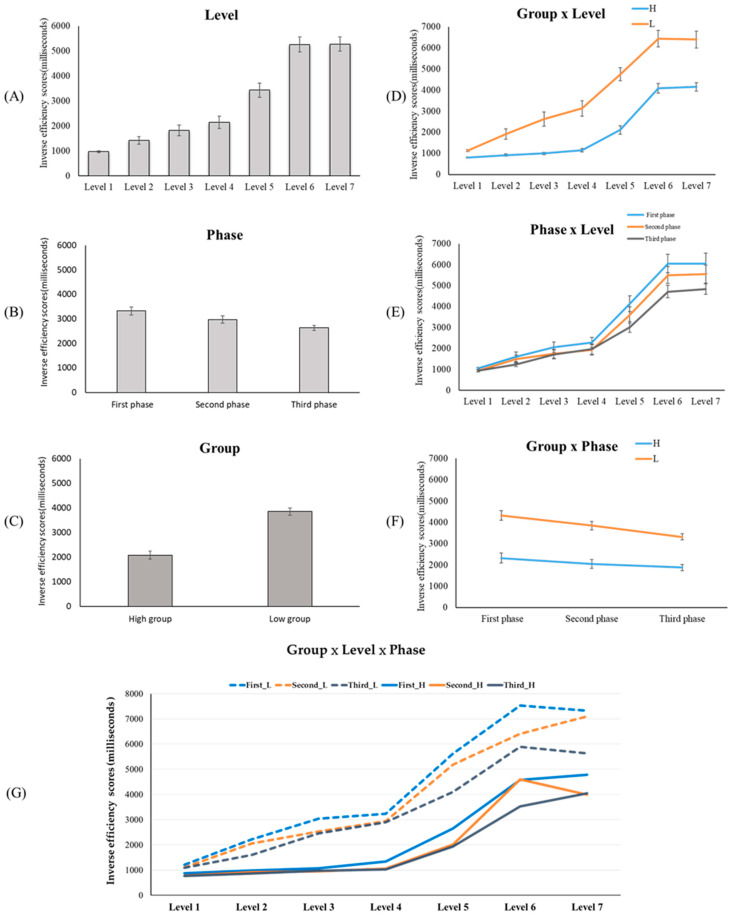
Results of three main effects (**A**–**C**), two-way interaction (**D**–**F**), and three-way interaction (**G**). (**G**) refers to the high- and low-efficiency groups’ IES of answering seven difficulty levels in three phases. The solid lines represent the IES of the high-efficiency group, and the dotted lines represent the IES of the low-efficiency group. The three phases are represented by different colors (first = blue line, second = orange line, and third = gray line).

We conducted a mixed three-way ANOVA (Figure 2); the between-subject factor was G (HE, LE), and the within-subject factors were L (L1 to L7) and P (1st, 2nd, 3rd).

#### 3.1.1. The Main Effects 

The main effect of G was significant (F = 52.706, *p* < 0.001, $\eta_{p}^{2}$ = 0.581), HE (M = 2081.93 ms, SE = 231.37 ms) showed a shorter IES than the LE (M = 3856.51 ms, SE = 92.41 ms). The main effect of Level was also significant (F = 137.173, *p* < 0.001, $\eta_{p}^{2}$ = 0.783). The post hoc tests are revealed in Table 2. The main effect of P was also significant (F = 15.500, *p* < 0.001, $\eta_{p}^{2}$ = 0.290). The post hoc tests revealed that the IES of 2nd was significantly shorter than the 1st (*p* < 0.05); the IES of 3rd was significantly shorter than the 1st (*p* < 0.05); and the IES of 3rd was also significantly shorter than 2nd (*p* < 0.05; 1st: M = 3316.88 ms, SE = 164.42 ms; 2nd: M = 2962.85 ms, SE = 149.76 ms; 3rd: M = 2627.93 ms, SE = 103.27 ms).

#### 3.1.2. Interactions and Simple Main Effects

The results of three two-way interactions (L × G, L × P, and G × P) and a three-way interaction (G × P × L) were shown in Table 2. The results showed a significant interaction between L and G (F = 7.118, *p* < 0.001, $\eta_{p}^{2}$ = 0.158), and a significant interaction between L and P (F = 2.539, *p* = 0.003, $\eta_{p}^{2}$ = 0.063). There was no significant interaction between G and P, and there was no significant interaction among the three factors (G, P, and L).

##### Two-Way Mixed Interaction (L × G) and Simple Main Effects

The results of the simple main effects in the within-subject factor (L) were shown in Table 2. The simple main effect of the HE condition was significant (F = 53.940, *p* < 0.001). The results of the post hoc were shown in Table 2 (L1: M = 802.32 ms, SE = 33.15 ms; L2: M = 920.70 ms, SE = 57.74 ms; L3: M = 998.43 ms, SE = 49.27 ms; L4: M = 1151.53 ms, SE = 89.13 ms; L5: M = 2110.37 ms, SE = 203.59 ms; L6: M = 4081.84 ms, SE = 231.66 ms; L7: M = 4152.70 ms, SE = 204.85 ms).

The simple main effect of the LE condition was significant (F = 103.389, *p* < 0.001). The results of post hoc were shown in Table 2 (L1: M = 1131.64 ms, SE = 52.80 ms; L2: M = 1916.65 ms, SE = 246.33 ms; L3: M = 2626.59 ms, SE = 333.86 ms; L4: M = 3129.87 ms, SE = 359.20 ms; L5: M = 4750.22 ms, SE = 313.59 ms; L6: M = 6433.49 ms, SE = 395.64 ms; L7: M = 6399.83 ms, SE = 401.02 ms).

The results of the simple main effects in the between-subject factor (G) were shown in Table 2. The simple main effect of L1 was not significant. The simple main effect of L2 was significant (F = 7.967, *p* = 0.005), the IES of the HE was shorter than the LE (HE: M = 920.70 ms, SE = 57.74 ms; LE: M = 1916.65 ms, SE = 246.33 ms). The simple main effect of L3 was significant (F = 21.292, *p* < 0.001), the IES of the HE was shorter than the LE (HE: M = 998.43 ms, SE = 49.27 ms; LE: M = 2626.59 ms, SE = 333.86 ms). The simple main effect of L4 was significant (F = 31.436, *p* < 0.001), the IES of the HE was shorter than the LE (HE: M = 1151.53 ms, SE = 89.13 ms; LE: M = 3129.87 ms, SE = 359.20 ms). The simple main effect of L5 was significant (F = 55.973, *p* < 0.001), the IES of the HE was shorter than the LE (HE: M = 2110.37 ms, SE = 203.59 ms; LE: M = 4750.22 ms, SE = 313.59 ms). The simple main effect of L6 was significant (F = 44.418, *p* < 0.001), the IES of the HE was shorter than the LE (HE: M = 4081.84 ms, SE = 231.66 ms; LE: M = 6433.49 ms, SE = 395.64 ms). The simple main effect of L7 was significant (F = 40.558, *p* < 0.001), the IES of the HE was shorter than the LE (HE: M = 4152.70 ms, SE = 204.85 ms; LE: M = 6399.83 ms, SE = 401.02 ms).

##### Two-Way Repeated Interactions (L × P) and Simple Main Effects

The results of the simple main effects in the level factor (L) were shown in Table 2. The simple main effect of 1st was significant (F = 79.404, *p* < 0.001). The simple main effect of 2nd was significant (F = 64.039, *p* < 0.001). The simple main effect of 3rd was significant (F = 39.429, *p* < 0.001). Please see Table 2 for more post hoc details on these three conditions.

The results of the simple main effects in the phase factor (P) were shown in Table 2. There were no simple main effects of L1, L2, L3, L4, and L5. The simple main effect of L6 was significant (F = 6.993, *p* = 0.001), the post hoc tests revealed that the IES of 3rd was shorter than 1st and 2nd, but there was no significant difference between 1st and 2nd (1st: M = 6054.32 ms, SE = 436.37 ms; 2nd: M = 5499.37 ms, SE = 400.79 ms; 3rd: M = 4708.51 ms, SE = 292.04 ms). The simple main effect of L7 was significant (F = 5.726, *p* = 0.004), the post hoc tests revealed that the IES of 3rd was shorter than 1st (1st: M = 6053.30 ms, SE = 495.59 ms; 3rd: M= 4835.01 ms, SE = 241.43 ms). 

### 3.2. A Mixed Two-Way ANOVA (G × L)

**Figure 3:**
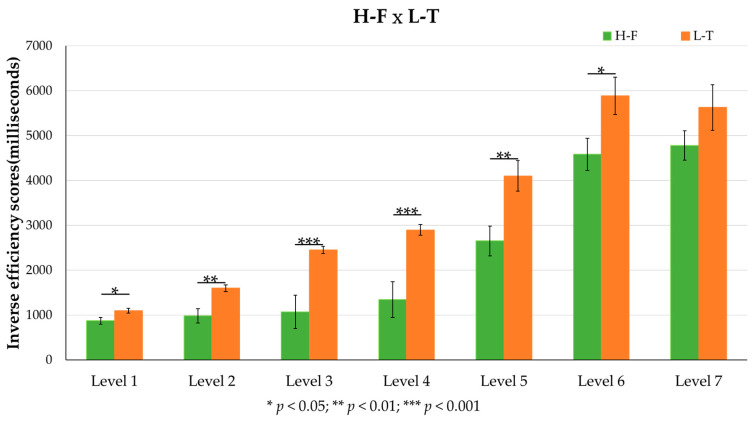
Inverse efficiency scores of the high-efficiency group in the first phase (H-F) and low-efficiency group in the third phase (L-T). More details of H-F and L-T showed in Table 3.

To further understand the practice effect, we conducted a mixed two-way ANOVA with G (H-F, L-T) as the between-subject factor, and L (L1 to L7) as the within-subject factor (Figure 3). 

The main effect of G was significant (F = 19.830, *p* < 0.001, $\eta_{p}^{2}\;$= 0.343), the H-F showed a shorter IES than the L-T (H-F: M = 2325.3 ms, SE = 145.02 ms; L-T: M = 3378.97 ms, SE = 186.97 ms).

The main effect of L was significant (F = 95.910, *p* < 0.001, $\eta_{p}^{2}\;$= 0.716). The post hoc tests revealed that there was no significant difference between L6 and L7, and there was also no significant difference between L2 and L3. However, the IES of L6 and L7 were significantly longer than other Levels. The IES of L5 was also significantly longer than L1, L2, L3, and L4. The IES of L4 was also significantly longer than L1, L2, and L3. The IES of L2 and L3 were significantly longer than L1 (L1: M = 982.57 ms, SE = 44.14 ms; L2: M = 1288.74 ms, SE = 86.55 ms; L3: M = 1760.79 ms, SE = 185.42 ms; L4: M = 2119.17 ms, SE = 202.11 ms; L5: M = 3377.34 ms, SE = 231.64 ms; L6: M = 5233.47 ms, SE = 269.96 ms; L7: M = 5202.73 ms, SE = 293.84 ms). The interaction between G and L was not significant.

## 4. Discussion

This section discusses four main questions.

### 4.1. Discussion of the Cognitive Load in a Computer-Based Testing Environment from the Perspective of Experimental Material Difficulty

Section 3.1 revealed that participants’ IES increased with the difficulty of the experimental materials, which suggested their increased cognitive load as the element interactivity was higher. These results concur with the argument of cognitive-load theory—the more difficult the learning material is, the higher the cognitive load becomes [16,17,18]. In addition, the results also showed their cognitive load to be a rapidly increasing pattern. When answering test items with difficulty levels of 1–4, students experienced a low cognitive load. The participants’ cognitive load increased rapidly when answering test items with a difficulty level of 5, and when answering test items with a difficulty level of 6–7, the participants experienced a high cognitive load. The aforementioned trend was observed in both high- and low-efficiency groups, which suggested that the pattern of increase in the two groups was consistent and that there would be no pattern differences due to efficiency (e.g., a slow increase in the high-efficiency group and rapid increase in the low-efficiency group). Test items involving four or fewer calculation steps could be easily solved by learners, whereas those involving five or more calculation steps required higher cognitive ability, particularly working memory.

**Figure A1:**
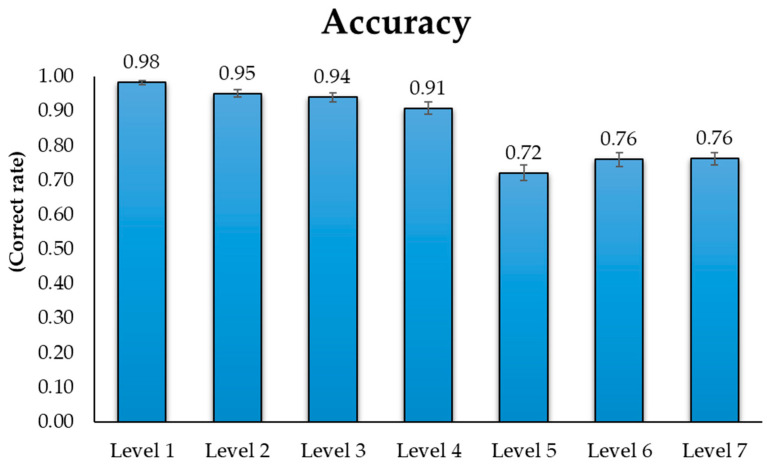
Accuracy of each level in this study.

Theoretically, if an examination time is sufficient and the examination mechanism allows for the use of pen and paper for calculation, college and graduate students should be able to answer all test items correctly considering that these algebra equations target fifth to seventh graders. However, from the accuracy, they did not correctly answer all mathematical problems—the accuracy rate for test items with difficulty levels of 1–4 and 5–7 was, respectively, ≥90% and approximately 70% (see Appendix A, Figure A1). Their failure to do so implied that their performance in computer-based testing might have been greatly influenced by their working memory [25,34]. Perhaps the computer-based testing environment interfered with students’ test taking, and the more difficult the test items were, the higher the interference became. From the administration of computer-based testing, the rigorous test-taking mechanism in this study may affect students’ performance, because each test item was only presented for 4.5 s and students must respond in 9 s (Figure 1C)—this requires a lot of working memory. In other words, compared with computer-based testing, people taking traditional paper-based testing can write their calculation processes on paper with a pen to reduce the cognitive load, which can be more effectively allocated for other tasks to improve test performance. Accordingly, the study results suggest that, when designing computer-based mathematics tests, attention should be paid to the difficulty of test items, which may result in the underestimation of the examinees’ performance. 

### 4.2. Discussion of the Cognitive Load of the High- and Low-Efficiency Group in a Computer-Based Testing Environment

According to analysis results, the high- and low-efficiency groups exhibited no difference when solving Level-1 test items but demonstrated increasing differences in processing efficiency as the test items became more difficult (Figure 2D). That is, the higher the difficulty level was, the more differences were observed between the two groups, with the high-efficiency group demonstrating lower IES. This finding suggested that the high-efficiency group had greater working memory capacity; thus, their performance would be higher in a computer-based testing environment requiring higher participants’ cognitive processing ability [35]. By contrast, the working-memory capacity of the low-efficiency group may be small and insufficient to cope with such a computer-based testing environment. Therefore, the disparity between the two groups gradually increased with the difficulty of the test items.

### 4.3. Discussion of the Cognitive Load from the Perspective of Practice Effect

The results revealed that the participants’ IES gradually decreased as the experiment progressed, and showed the lowest in the third phase (Figure 2B,E). This indicated that students exhibit the practice but not the fatigue effect when solving more mathematical problems, and practice was effective in reducing the participants’ cognitive load. To sum up, the existence of the practice effect denotes: first, learners’ problem-solving efficiency increases with experience; second, learning automation; third, that the most crucial characteristic is cognitive load reduction [27]. This observation indicates that adding phase design to computer-based testing helps to examine the cognitive load change of learners in a computer-based testing environment over time.

### 4.4. Can Practice Effect Reduce the Disparity between High- and Low-Efficiency Groups?

According to the findings (Figure 3), practice can help reduce cognitive load. Another concern is if the low-efficiency group’s test performance in the third phase can be comparable to or better than the high-efficiency group’s test performance in the first phase. To address this question, we performed an analysis (Figure 3) and found that the disparity still existed—the test performance of the high-efficiency group in the first phase was still superior to that of the low-efficiency group in the third phase. Therefore, the cognitive ability of the high-efficiency group may be initially superior to that of the low-efficiency group. This enables the high-efficiency group to better adapt to the computer-based testing environment. The low-efficiency group may need more practice in the computer-based testing environment to support their cognitive load; therefore, more functional designs may be required when implementing computer-based testing to reduce the influence of a high cognitive load on students’ test performance. For example, notes, handwriting, drawing, or other methods of record-keeping may be incorporated to reduce cognitive load in the computer-based testing environment [36,37].

## 5. Limitations and Future Work

Future studies should clarify the impact of the digital environment on students by discussing the relationship between the test item presentation time and the cognitive load. Although the algebra equations selected in this study were defined as learning materials with high element interactivity according to cognitive-load theory, they still remain relatively basic in the field of mathematics. Higher-level algebra problems can be used in future relevant studies. In addition, investigating the algebraic concepts of the participants can help to provide more information.

## 6. Conclusions

Regarding the cognitive load of students answering mathematics algebra questions in a computer-based testing environment, IES was used as an indicator of cognitive load, and three perspectives, element interactivity, practice effect, and individual differences were analyzed in this study. There are four main findings: First, the cognitive load increased rapidly but not slowly with the higher element interactivity. Second, the cognitive load of the high-efficiency group was indeed lower than the low-efficiency group, showing the high-efficiency group has an advantage compared to the low-efficiency group in the computer-based testing environment. Third, “practice” shows a significant effect in reducing cognitive load, especially in the test items of level 6 and level 7. Fourth, the cognitive load of the low-efficiency group in the third phase was still higher than the high-efficiency group in the first phase, showing that the low-efficiency group can reduce but not equalize the disparity with the high-efficiency group through practice three times; the low-efficiency group may need more practice in the computer-based testing environment to support their cognitive load.

## Figures and Tables

**Table 1:** Materials.

Levels	Level 1	Level 2	Level 3	Level 4	Level 5	Level 6	Level 7
Calculation steps	1	2	3	4	5	6	7
Types	Unary linear equation		Simultaneous linear equations				
Example	□ + 2 = 3	3 × □ + 5 = 14	$\{\begin{array}{c} x+y=2\\ 2x+y=3 \end{array}$	$\{\begin{array}{c} x+y=8\\ 3x+y=18 \end{array}$	$\{\begin{array}{c} x+3y=34\\ 5x+3y=62 \end{array}$	$\{\begin{array}{c} 4x+8y=76\\ 6x+3y=51 \end{array}$	$\{\begin{array}{c} 7x-6y=-4\\ 6x-7y=\;4 \end{array}$

**Table 2:** Results of mixed three-way ANOVA.

Main Effects	F	Post Hoc		
G	52.706 ***	HE < LE		
P	15.500 ***	1st > 2nd; 1st > 3rd; 2nd > 3rd		
L	137.173 ***	(1) L1 < L2; L1 <L3; L1 < L4; L1 < L5; L1 < L6; L1 < L7 (2) L2 < L4; L2 < L5; L2 < L6; L2 < L7 (3) L3 < L5; L3 < L6; L3 < L7 (4) L4 < L5; L4 < L6; L4 < L7 (5) L5 < L6; L5 < L7		
Interaction	F	Simple Main Effects		
		Effects	F	Post Hoc
L × G	7.118 ***	L		
		HE	53.940 ***	L1 < L3; L1 < L4; L1 < L5; L1 < L6; L1 < L7 L2 < L5; L2 < L6; L2 < L7 L3 < L5; L3 < L6; L3 < L7 L4 < L5; L4 < L6; L4 < L7 L5 < L6; L5 < L7
		LE	103.389 ***	L1< L2; L1 < L3; L1 < L4; L1 < L5; L1 < L6; L1 < L7 L2 < L4; L2 < L5; L2 < L6; L2 < L7 L3 < L4; L3 < L5; L3 < L6; L3 < L7 L4 < L5; L4 < L6; L4 < L7 L5 < L6; L5 < L7
		G		
		L1	0.871	
		L2	7.967 **	HE < LE
		L3	21.292 ***	HE < LE
		L4	31.436 ***	HE < LE
		L5	55.973 ***	HE < LE
		L6	44.418 ***	HE < LE
		L7	40.558 ***	HE < LE
L × P	2.539 **	L		
		1st	79.404 ***	L1 < L3; L1 < L4; L1 < L5; L1 < L6; L1 < L7 L2 < L4; L2 < L5; L2 < L6; L2 < L7 L3 < L5; L3 < L6; L3 < L7 L4 < L5; L4 < L6; L4 < L7 L5 < L6; L5 < L7
		2nd	64.039 ***	L1 < L2; L1 <L3; L1 < L4; L1 < L5; L1 < L6; L1 < L7 L2 < L5; L2 < L6; L2 < L7 L3 < L5; L3 < L6; L3 < L7 L4 < L5; L4 < L6; L4 < L7 L5 < L6; L5 < L7
		3rd	39.429 ***	L1 < L2; L1 <L3; L1 < L4; L1 < L5; L1 < L6; L1 < L7 L2 < L4; L2 < L5; L2 < L6; L2 < L7 L3 < L5; L3 < L6; L3 < L7 L4 < L5; L4 < L6; L4 < L7 L5 < L6; L5 < L7
		P		
		L1	0.058	
		L2	0.739	
		L3	0.543	
		L4	0.601	
		L5	4.771	
		L6	6.993 **	1st > 3rd; 2nd > 3rd
		L7	5.726 *	1st > 3rd
G × P	1.989			
G × P × L	1.361			

* *p* < 0.05; ** *p* < 0.01; *** *p* < 0.001; G = group factor; P = phase factor; L = level factor; HE = high-efficiency; LE = low-efficiency; 1st = first phase; 2nd = second phase; 3rd = third phase; L1 = Level 1; L2 = Level 2; L3 = Level 3; L4 = Level 4; L5 = Level 5; L6 = Level 6; L7 = Level 7.

**Table 3:** The mean inverse efficiency scores of the high- and low-efficiency groups (unit: ms).

Group-Phase ^1^	Level 1		Level 2		Level 3		Level 4		Level 5		Level 6		Level 7	
	Mean	SE	Mean	SE	Mean	SE	Mean	SE	Mean	SE	Mean	SE	Mean	SE
H-F	871.0	53.2	980.3	71.2	1070.8	74.3	1342.2	115.6	2652.9	332.8	4581.0	408.7	4778.7	495.0
H-S	778.6	43.1	918.6	83.7	954.7	51.7	1055.1	89.4	2008.6	248.7	4596.5	414.9	3993.4	285.9
H-T	762.8	46.5	866.9	70.9	970.5	64.1	1030.6	109.3	1933.2	222.9	3531.1	282.4	4043.2	270.2
L-F	1211.4	66.2	2212.2	402.3	3033.5	372.0	3230.5	368.0	5616.4	471.3	7527.6	621.4	7327.9	769.6
L-S	1106.8	68.6	2058.3	316.8	2532.4	333.9	2943.2	402.0	5178.6	530.2	6402.2	633.6	7100.2	647.4
L-T	1094.2	70.5	1597.2	157.8	2450.8	363.3	2896.1	387.3	4101.8	322.3	5885.9	352.8	5626.8	316.7

^1^ Group-Phase: H = high-efficiency group, L = low-efficiency group; F = first phase, S = second phase, T = third phase.

## Data Availability

The data used in the current research are available from the corresponding author upon reasonable request.

## Appendix A

The largest gap (about 19%) was presented between Level 4 and Level 5. The accuracy of the higher levels (Level 5–7) was over 70% at least (the chance level is 50%).
